# Incidental Diagnosis of Oligosymptomatic Bilateral Perirenal Erdheim–Chester Disease during Emergency Investigation for COVID-19 Infection

**DOI:** 10.1155/2023/4683188

**Published:** 2023-06-03

**Authors:** Juliano Cordova Vargas, Caio Cardozo, Renata Stanzione, Lucas Fiore, Felipe D'Almeida Costa, Rodrigo Fonseca Abreu, Nelson Hamerschlak, Guilherme Perini

**Affiliations:** ^1^Hematology Department, Americas Oncologia e Hematologia, São Paulo, SP, Brazil; ^2^Hematology Department, Hospital Samaritano Higienópolis, São Paulo, SP, Brazil; ^3^Hematology Department, Hospital Metropolitano da Lapa, São Paulo, SP, Brazil; ^4^School of Medicine, Centro Universitário São Camilo, São Paulo, SP, Brazil; ^5^Hematology Department, Hospital Israelita Albert Einstein, São Paulo, SP, Brazil; ^6^Radiology Department, Hospital Metropolitano da Lapa, São Paulo, SP, Brazil; ^7^Radiology Department, Hospital Samaritano Higienópolis, São Paulo, SP, Brazil; ^8^Pathology Department, A. C. Camargo Cancer Center, São Paulo, SP, Brazil

## Abstract

Erdheim–Chester disease (ECD), a rare form of non-Langerhans histiocytosis, is a multisystem disorder. The case reported here refers to a 49-year-old man presenting at the emergency room with respiratory symptoms. While undergoing diagnostic tests for COVID-19 infection, tomography revealed asymptomatic bilateral perirenal tumors, while renal function remained unaltered. ECD was suggested as an incidental diagnosis and confirmed by core needle biopsy. This report provides a brief description of the clinical, laboratory, and imaging findings in this case of ECD. This diagnosis, albeit rare, should be taken into consideration in the context of incidental findings of abdominal tumors to ensure that treatment, when required, is instituted early.

## 1. Introduction

Histiocytosis refers to a heterogeneous group of rare proliferative disorders in which histiocytes, i.e., bone marrow-derived macrophages, are present in tissues. This group of diseases is classified according to their biological behavior and the phagocytic cell system involved [1–3]. Histiocytic and dendritic cell neoplasms originate from or show differentiation towards cells of the mononuclear phagocyte system, which consists of macrophages, monocytes, and dendritic cells. Malignant conditions include Erdheim–Chester disease (ECD), histiocytic sarcoma, Rosai–Dorfman disease, and anaplastic lymphoma kinase- (ALK-) positive histiocytosis [4]. The revised classification of histiocytosis and neoplasms of the macrophage-dendritic cell lineages [5] includes five groups of diseases: Langerhans-related, cutaneous and mucocutaneous, malignant histiocytosis, Rosai–Dorfman disease, and hemophagocytic lymphohistiocytosis as well as macrophage activation syndrome. ECD is part of the Langerhans-related group that also includes Langerhans cell histiocytosis (LCH), mixed LCH/ECD, and indeterminate cell histiocytosis [5].

The Histiocyte Society classification currently divides histiocytic disorders into five categories based on clinical, histologic, immunophenotypic, and molecular features [2]. In that classification, the Langerhans (L) group includes LCH, ECD, mixed LCH/ECD, indeterminate cell histiocytosis, and extracutaneous juvenile xanthogranuloma. The World Health Organization (WHO) provided a classification of histiocytic and dendritic cell neoplasms in its 2017 classification of hematologic malignancies but did not classify nonmalignant disorders. The WHO classification is divided into ten categories: histiocytic sarcoma, LCH, Langerhans cell sarcoma, indeterminate dendritic cell tumor, interdigitating dendritic cell sarcoma, follicular dendritic cell sarcoma, inflammatory pseudotumor-like follicular/fibroblastic dendritic cell sarcoma, fibroblastic reticular cell tumor, disseminated juvenile xanthogranuloma, and ECD.

ECD, a rare form of non-LCH, is a multisystem disorder that was first described in 1930 by Erdheim and Chester. Since then, fewer than 1,000 cases have been reported in the literature [1]. The incidence of ECD is unknown; however, according to reports, men are more frequently affected at a ratio of 3 : 1. The median age of affected individuals is 50 to 60 years, and although the condition seldom affects the pediatric population, rare cases have been reported in children under 15 years of age [1, 6]. The disease is now understood as a clonal disorder involving somatic mutations such as the mitogen-activated protein kinase (MAPK) in addition to other, already well-described, molecular alterations such as mutations of the BRAF gene. The inflammatory state contributes to disease progression and consequent tissue damage [3].

This report refers to a man presenting with respiratory symptoms, fever, and suspected SARS-CoV-2 virus infection in whom ECD in its asymptomatic form was detected incidentally. In a rare presentation, bilateral perirenal involvement was found, with a high tumor burden despite the fact that kidney function remained unimpaired.

## 2. Case Presentation

A 49-year-old man was admitted to the Hospital Metropolitano Lapa in the Brazilian city of São Paulo on November 3, 2020. The patient complained of asthenia over the preceding 15 days together with a fever that peaked at 38.5°C and a nonproductive cough. Polymerase chain reaction for COVID-19, performed on the same day, was negative. The patient reported no weight loss, night sweats, neurological complaints, and abdominal or bone pain. He had a history of recurrent nephrolithiasis previously treated surgically. He was under no continuous medication. According to the patient, there was no family history of any hematologic malignancies. At physical examination, there was no sign of palpable adenomegaly or visceromegaly.

Laboratory tests revealed a hemoglobin level of 14 g/dl, mean corpuscular volume: 83 fl, total leukocytes: 7,900 WBCs per microliter of blood, with 2,500 neutrophils per microliter, and 255,000 platelets per microliter. The erythrocyte sedimentation rate was 22 mm (reference value ≤ 30 mm). Clinical chemistry parameters (reference values in brackets) showed normal levels for lactic dehydrogenase: 151 IU/L (≤220 IU/L), beta 2 microglobulin: 1.7 g/ml (≤2.5 g/ml), creatinine: 0.9 mg/dL (≤1.3 mg/dL), urea: 25 mg/dL (≤50 mg/dL), AST: 25 U/L (≤40 U/L), ALT: 28 U/L (≤56 U/L), gamma-glutamyl transferase: 45 U/L (≤60 U/L), serum alkaline phosphatase: 75 U/L (≤150 U/L), albumin: 3.5 mg/dL (≤5.2 mg/dL), and uric acid: 2.5 mg/dL (≤7 mg/dL).

**Figure 1:**
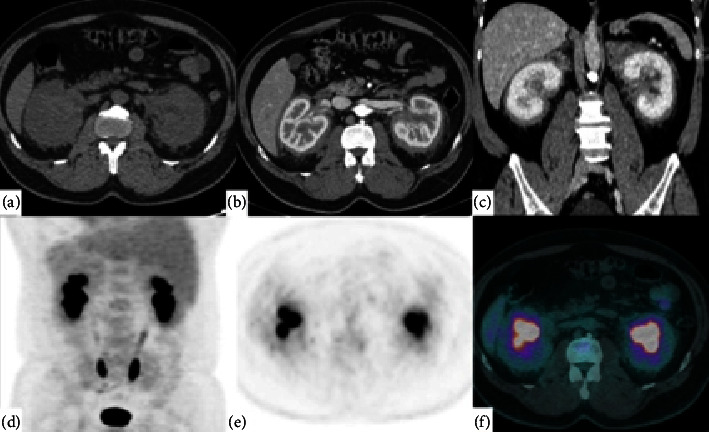
Computed tomography images of the abdomen: (a) before administration of the intravenous iodinated contrast, (b) corticomedullary phase, and (c) coronal reformatted image of the nephrographic phase, showing tissue with soft-tissue density surrounding the renal parenchyma and infiltrating the renal pelvis bilaterally. Note the slight dilatation of the bilateral collecting system resulting from extrinsic compression by the tissue. Positron emission tomography/computed tomography (PET-CT): no radiopharmaceutical uptake is seen on PET-CT as shown in (d) coronal PET, (e) axial PET, and (f) fusion of PET with tomographic image. Percutaneous biopsy confirmed the hypothesis of renal histiocytosis (Erdheim–Chester disease).

Computed tomography (CT) at admission revealed a bilateral paravertebral formation with a soft tissue component, occupying bilateral perirenal spaces (Figures 1(a)–1(c)). The size and shape of the kidneys were normal, with slight dilatation of the adjacent pyelocaliceal system resulting from extrinsic compression by the tissue. The possibility of a malignant condition such as lymphomas, germ tumors, or histiocytosis was then considered.

**Figure 2:**
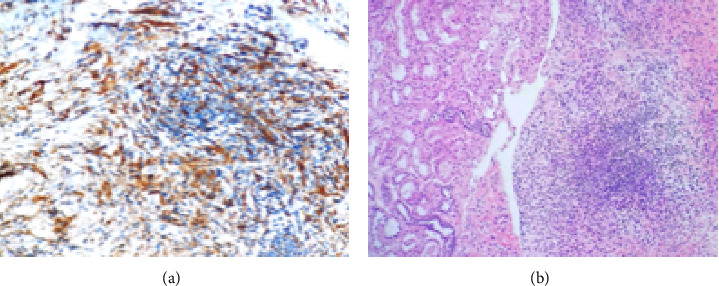
Photomicrography. CT-guided core biopsy of the perirenal lesion. (a) Fibrous tissue with infiltrate of plasma cells, lymphocytes, and histiocytes (right) adjacent to renal parenchyma (left) (HE, 40x). (b) Immunohistochemical staining for CD68, highlighting the histiocytic infiltrate. CT: computed tomography.

On November 6, a CT-guided core biopsy of the perirenal lesion was performed (Figure 1), with histological results revealing fibrous tissue with infiltrate of plasma cells, lymphocytes, and histiocytes adjacent to renal parenchyma (Figure 2(a)**)**. In addition, immunohistochemical staining for CD68 highlighted a histiocytic infiltrate (Figure 2(b)). These findings were compatible with that of ECD. The patient was discharged from hospital the following day.

On November 8, he underwent positron emission tomography/CT (PET-CT), which showed no uptake in the bilateral perirenal and pararenal regions with a standardized uptake value of 3, no delimitation of the cleavage plane between the kidneys and adrenals and some ectasia of the pyelocaliceal system (Figures 1(d)–1(f)).

Since the patient had few symptoms, watchful waiting was the preferred approach and no treatment was instituted. At his most recent follow-up visit in October 2022, the patient was asymptomatic with preserved renal function. CT performed during follow-up showed that the perirenal mass remained stable and there were no new lesions at any other sites.

## 3. Discussion

ECD is a clonal disorder resulting from myeloid precursors in the bone marrow. The somatic BRAF mutation is a common finding, together with a significant increase in inflammatory cytokines. Mutations activating the MAPK protein are found in over 80% of patients with ECD, principally BRAF V600E (57–70% of cases) followed by MAP2K1 (in around 20% of cases). Most cases have a gain-of-function somatic alteration activating the MAPK cell signaling pathway. Unlike other forms of non-LCH such as Rosai–Dorfman disease and juvenile xanthogranuloma, with ECD the BRAF p.V600E mutation is present in 50–60% of cases [7]. Even patients without the BRAF p.V600E mutation frequently have other mutations that also activate the MAPK pathway such as those in BRAF, ARAF, NRAS, KRAS, or MAP2K1 [8]. There may also be mutations in the PIK3CA oncogene (p.E542K, p.E545K, p.A1046T, and p.H1047R), and when present, these are associated with BRAF mutations in most cases [9]. Durham et al. found mutations in MAPK signaling intermediates in CD34+ cells from patients with ECD. Those data indicate that some cases of ECD can result from the accumulation of histiocytes in tissue as a consequence of mutated bone marrow progenitors [10]. Notwithstanding, some cases of ECD can occur in the absence of clonal hematopoiesis [11].

Mutations in MAP2K1, which encodes MEK1, are present in 20–25% of cases of LCH. Immunohistochemistry shows activation of the MAPK/ERK pathway. In one study, expression of cyclin D1 (which lies downstream of the MAPK/ERK signaling pathway) by Langerhans cells was demonstrated in all 39 cases of LCH evaluated. Phospho-ERK, an indication of pathway activation, was detected in all but one of these cases. MAPK alterations have also been reported in over 80% of single-system pulmonary LCH, with the frequency of BRAF V600E being between 28 and 36% [12–14]. Malignant Langerhans cells originate from myeloid dendritic cells, as shown by gene expression data and mutation tracking. Gene expression array data have shown that they originate from bone marrow-derived myeloid cells rather than from the epidermal Langerhans cells that they resemble morphologically [15]. Their myeloid origin was confirmed after BRAF V600E was found in subsets of dendritic cells, mature monocytes, committed myeloid progenitors, and CD34+ cells from affected LCH patients [10, 16].

In the case of ECD, there are no infectious disorders etiologically related to the disease; however, inflammatory status could contribute to disease progression and tissue damage [1, 3]. The disease can affect the body systemically, with involvement of the central nervous system, skin, bones, lungs, retroperitoneum, mediastinum, and eyes. Bone involvement is the most common finding at diagnosis (bone pain is present in 26% of cases), associated with at least one other non-bone-related form of involvement such as neurological symptoms (23%), diabetes insipidus (22%), and constitutional symptoms (20%). In some cases, there is cardiovascular involvement, with morbidity and mortality being greater in such cases. Cardiovascular conditions include changes to the heart conduction system, heart valve abnormalities, and periaortic fibrosis. When the central nervous system is affected, involvement may be intra-axial or extra-axial, which may imply oligosymptomatic or even neurodegenerative and limiting conditions [1, 15]. Renal findings of a “hairy kidney” are relatively common, together with hydronephrosis and progressive deterioration of renal function [1].

In asymptomatic patients, ECD may be found incidentally during routine imaging tests [15, 17]. In the case reported here, the diagnosis was made incidentally in an asymptomatic patient who sought care for other clinical complaints and suspected COVID-19 infection. Despite the finding of an image of soft tissue surrounding the renal parenchyma bilaterally, renal function was not impaired.

ECD is suspected when clinical and radiological findings are compatible with the disease and the suspected diagnosis is then confirmed histologically. Typical radiology findings in the abdomen include retroperitoneal involvement on CT due to the presence of masses involving the soft tissues and the psoas muscle imprisoning the perirenal fat [15], as found in the case described here. Biopsy is mandatory for diagnosis because, in addition to providing histological confirmation, it also allows molecular studies to be performed, which are fundamental when making decisions regarding treatment. Samples taken from cutaneous and perirenal lesions are preferable as they contain a high concentration of histiocytes compared to other sites such as bone lesions [15, 17].

Histologically, ECD is characterized by tissue infiltration of foamy, lipid-laden and/or small mononuclear histiocytes. Variable proportions of Touton giant cells, small lymphocytes, plasma cells, and/or neutrophils can be seen. Interestingly, admixed LCH may be present in the same biopsy specimen. A variable degree of fibrosis is present and can be predominant, resulting in misinterpretation of the lesion as a reactive fibroinflammatory process. However, other histological patterns have also been described [4, 15, 18–20]. In the current case, fibrous tissue with prominent histiocytes, plasma cells and lymphocytic infiltrate was present. Immunohistochemistry showed positivity for CD68. Indeed, at immunohistochemistry, ECD samples show positivity for histiocytic markers such as CD68 and CD163 and negativity for CD1a and CD207, thus differentiating ECD from LCH [18–20]. In the case described here, molecular testing for BRAF V600E was performed, with results being negative. No further molecular testing was available. In view of the clinical presentation and typical findings at histology, ECD was the presumed diagnosis.

Differential diagnoses include not only LCH, but also Paget's disease, polyneuropathy, organomegaly, endocrinopathy, myeloma protein and skin changes (POEMS) syndrome, Rosai–Dorfman disease, and hemophagocytic lymphohistiocytosis. Clinical management depends on the manifestations of the condition and their severity. One option is to follow the patient up with regular clinical visits and laboratory tests, but with no pharmaceutical treatment; however, chemotherapy can be initiated with regimens that include interferon, corticosteroids, methotrexate, cladribine, trametinib, and ALK inhibitors [15, 21].

For patients with BRAF mutations and an indication for treatment, targeted therapy with vemurafenib is recommended [21]. For those without a BRAF mutation, effective therapeutic options are fewer. The absence of symptoms, no impairment to kidney function, and a finding of no BRAF mutation at immunohistochemistry indicate the insidious nature of the disease. Therefore, regular clinical visits and laboratory follow-up every 4–6 months was the chosen approach in the case reported here. At the latest follow-up visit in October 2022, the patient had no new complaints and there was no organ dysfunction. Kidney function remained unaffected and the imaging tests were unchanged. These follow-up findings reinforced the decision to monitor the patient at regular clinical visits.

## 4. Conclusions

ECD is a rare entity, with a multifaceted presentation ranging from asymptomatic to severe clinical manifestations and organ involvement. Diagnosis may be incidental, as in the case described here in which the disease was detected during investigation for COVID-19 infection. Given the molecular profile and the absence of symptoms and organ involvement, no treatment was recommended. However, regular clinical, laboratory, and radiological follow-up of this patient is crucial.

## Data Availability

The data used to support the findings of this study are included within the article.
